# Pyrotinib plus capecitabine could significantly improve overall survival in HER2-positive metastatic breast cancer

**DOI:** 10.1038/s41392-023-01322-w

**Published:** 2023-03-19

**Authors:** Xiuwen Guan, Fei Ma, Qiao Li, Shanshan Chen, Ying Fan, Jiayu Wang, Yang Luo, Pin Zhang, Qing Li, Binghe Xu

**Affiliations:** Department of Medical Oncology and State Key Laboratory of Molecular Oncology, National Cancer Center/Cancer Hospital, Chinese Academy of Medical Sciences and Peking Union Medical College, Beijing, China

**Keywords:** Breast cancer, Drug development

**Dear Editor**,

The irreversible pan-ErbB tyrosine kinase inhibitors (TKIs), including neratinib and pyrotinib, demonstrated more complete inhibition towards ErbB -family and promising antitumor activity compared to lapatinib, a reversible TKI.^1^ In the phase III NALA study for HER2-positive metastatic breast cancer (MBC) who were progressed after two lines of HER2-targeted regimens,^2^ significant improvement was observed in progression-free survival (PFS) in patients receiving neratinib combined with capecitabine when compared to lapatinib plus capecitabine (L + C) group. However, the 2.2-month PFS improvement in neratinib plus capecitabine cohort failed to translate to a significant benefit in the overall survival (OS, 24.0 vs 22.2 months, *P* = 0.2086).^2^ While another irreversible TKI, pyrotinib combined with capecitabine (P + C) achieved clinically and statistically significant improvement in the PFS and a trend of benefits in the OS when compared to the L + C group, based on the interim analysis (with the cutoff of March 31, 2019) of the phase III PHOEBE study.^3^ Generally speaking, current evidence was still limited regarding the survival data of irreversible TKIs.

Thus, we summarized the updated individual patient-level data from the clinical trials in National Cancer Center of China for patients receiving pyrotinib plus capecitabine, to further investigate the OS of irreversible TKI in HER2-positive MBC. Individual patient-level data of the phase I study for P + C^4^ (NCT02361112), the pivotal phase II study^5^ (NCT02422199) and the PHOEBE phase III study^3^ (NCT03080805) was summarized and analyzed.^6^ Across the three clinical trials, female patients of HER2-positive MBC were assigned to receive pyrotinib 400 mg or lapatinib 1250 mg orally once per day for each 21-day cycle, combined with capecitabine (1000 mg/m^2^ orally twice per day on days 1–14). Original individual patient-level data was collected and analyzed regarding clinicopathological characteristics, and updated survival in this analysis. The data from all the enrolled participants including those excluded by the investigators in these three clinical trials was verified to decrease the potential bias.

Individual clinicopathological characteristics and survival data across the three trials were analyzed including 36 patients in the P + C cohort and 29 patients in the L + C cohort (Supplementary Fig 1). The baseline characteristics of age distribution, position of metastatic site at screening, hormone receptor (HR) status, previous chemotherapy, and prior trastuzumab treatment were well balanced between these two cohorts (Supplementary Table 1). In this study, the median duration of follow-up in PFS was 69.3 months (95% CI:24.0-114.5 months) and in OS was 69.3 months (95% CI:37.9–100.7 months). Death was reported in 19 (52.8%) participants in the P + C cohort and 22 (75.9%) participants in the L + C cohort. At the time of data cutoff (December, 2021), a total of seven patients were still receiving the treatment following the protocol in the trial cohorts and all of them were from the pyrotinib group, including two from the phase I trial, one from the pivotal phase II trial and four from the PHOEBE phase III trial.

The patients who received P + C were observed with significantly longer PFS [22.0 months (95% CI:7.1–36.9) vs 6.9 months (95% CI:4.0–9.7), *P* < 0.001) and OS [59.9 months (95% CI:29.3–90.6) vs 31.2 months (95% CI:27.0–35.5), *P* = 0.033) when compared to those with L + C (Fig. 1a, b). For patients with visceral metastases, consistent survival benefit trends in PFS and OS were observed with the whole population (median PFS, 22.1 vs 5.5 months, *P* < 0.001; median OS, 59.9 vs 30.8 months, *P* = 0.031, Fig. 1c, d). In the subgroup analysis of HR status, both HR-positive patients and HR-negative patients were observed with a pyrotinib benefit in PFS in the comparison between the cohort of P + C and those of L + C (for HR-positive MBC patients:19.2 vs 5.5 months, *P* < 0.001; for HR-negative MBC patients: 22.1 vs 6.9 months, *P* = 0.001, Fig. 1e, g). However, consistent results of OS benefit in pyrotinib combination group reached in HR-positive MBC patients (70.5 vs 27.0 months, *P* = 0.016, Fig. 1f), while in HR-negative subgroup, no significant difference was observed in the results of OS between these two cohorts (59.9 vs 43.8 months, *P* = 0.440, Fig. 1h). Regardless of prior trastuzumab treatment, significantly longer PFS was observed in the P + C cohort when compared to the L + C cohort (for patients with prior trastuzumab treatment, 16.5 vs 6.9 months, *P* < 0.001; for trastuzumab-naive patients, 27.5 vs 5.5 months, *P* = 0.004), while no significant difference reached in OS between these two cohorts (for patients with prior trastuzumab treatment, 47.1 vs 32.3 months, *P* = 0.170; for trastuzumab-naive patients, 59.9 vs 15.2 months, *P* = 0.104, Supplementary Fig. 2).

**Fig. 1:**
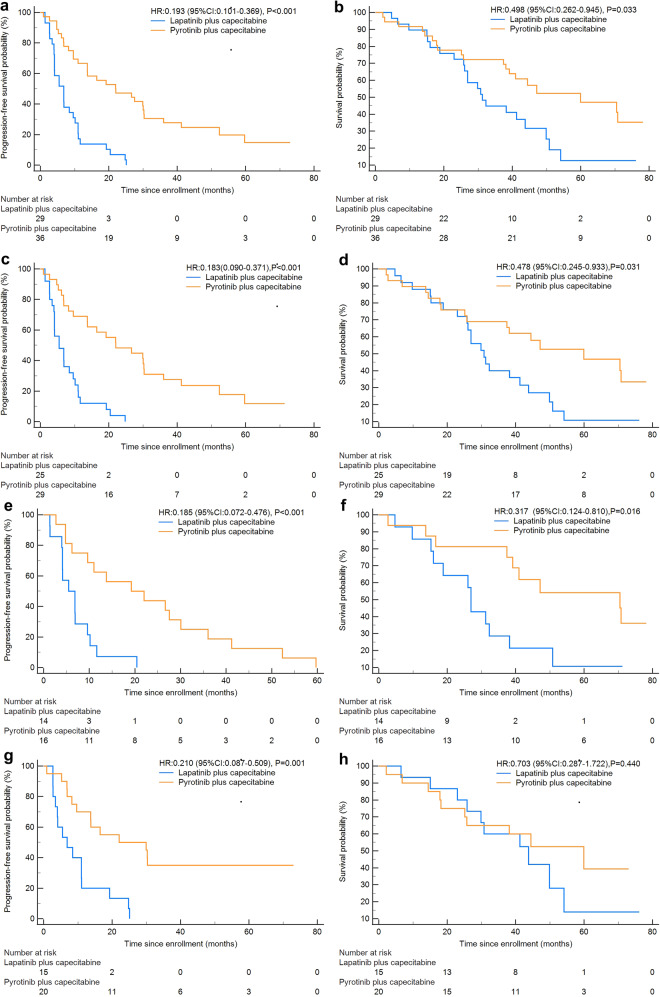
Kaplan–Meier analysis-based estimation of **a** PFS probability, and **b** OS probability for patients receiving P + C and L + C. Kaplan–Meier analysis-based estimation of **c** PFS probability and **d** OS probability for patients with visceral metastases receiving P + C and L + C. Kaplan–Meier analysis-based estimation of **e** PFS probability and **f** OS probability for HR-positive/HER2-positive patients receiving P + C and L + C. Kaplan–Meier analysis-based estimation of **g** PFS probability and **h** OS probability for HR-negative/HER2-positive patients receiving P + C and L + C

This analysis based on updated survival outcomes provided insight into the survival benefit of P + C in HER2-positive MBC. As a novel irreversible TKI that potently targets on EGFR /HER1, HER2, and HER4, pyrotinib demonstrated promising antitumor activity in HER2-positive MBC from the phase I to phase III studies.^3–5,7^ In the phase III NALA trial, the 2.2-month significantly PFS improvement (mean PFS 8.8 vs 6.6 months, *P* = 0.0003) in the neratinib plus capecitabine group failed to translate to a significant benefit in OS in comparison with the L + C group (24.0 vs 22.2 months; HR: 0.88; 95% CI:0.72–1.07; *P* = 0.2086). Nevertheless in this analysis, both statistically significantly improved PFS (22.0 vs 6.9 months, *P* < 0.001) and OS (59.9 vs 31.2 months, *P* = 0.033) were observed in those who received P + C when compared to the L + C arm, which appears to indicate pyrotinib-based regimens may be a promising treatment option for HER2-positive MBC. However, direct cross-trial comparisons could not be made on the basis of the limited sample size in this study and different enrolled criteria between these clinical trials.

In HER2-positive MBC patients, the standard second-line regimen is the antibody-drug conjugates (ADCs), including trastuzumab deruxtecan and trastuzumab emtansine. Regardless of the breakthroughs in durable antitumor activity and therapeutic response in ADCs for pretreated HER2-positive MBC, current data on survival outcomes was also limited. In the phase III EMILIA trial^8^ for HER2-positive MBC patients with prior treatment of trastuzumab and taxane, trastuzumab emtansine improved the OS when compared with the L + C group (29.9 vs 25.9 months, HR: 0.75; 95% CI 0.64–0.88). As for survival outcome of trastuzumab deruxtecan, the median OS was not reached at the time of reports for the phase II^9^ and phase III studies.^10^ Based on this pooled dataset, a comparable OS result of 59.9 months in P + C cohort was observed according to the median follow-up duration of 69.3 months. The results suggested that pyrotinib combination regimens might be an alternative option in the second-line treatment in regions where ADCs are not available, though direct comparisons could not be made between different clinical trials.

One of the main limitations of our study is related to the relatively small sample size. Besides that, the difference of the included/excluded criteria in these three clinical trials might result in a different population in these trials and limit the credibility of conclusions. Thus, no solid conclusion could be made from this pooled dataset of limited sample size and inevitable bias of cross-trial comparisons. Further final survival results from the PHOEBE phase III trial would probably bring us more evidence for survival outcome of irreversible pan-ErbB TKI.

In conclusion, the results based on individual patient-level data from clinical trials in National Cancer Center of China indicated that promising PFS and OS might be achieved in the treatment of pyrotinib plus capecitabine in HER2-positive MBC patients.

## Supplementary information

Supplementary Materials

Supplementary Figure 1

Supplementary Figure 2

## Data Availability

All the datasets analyzed are available to researchers via the corresponding author upon reasonable request.
