# Supplementary material for: Pyrotinib plus capecitabine could significantly improve overall survival in HER2-positive metastatic breast cancer
Source: Signal Transduct Target Ther. 2023 Mar 19;8:118. doi: 10.1038/s41392-023-01322-w (PMC10025258; doi:10.1038/s41392-023-01322-w)
Supplement: Supplementary file 1 — Supplementary Materials [file 41392_2023_1322_MOESM1_ESM.docx]

Supplementary Materials for

**Pyrotinib plus capecitabine could significantly improve overall survival in HER2-positive metastatic breast cancer**

Xiuwen Guan, Fei Ma, Qiao Li, Shanshan Chen, Ying Fan, Jiayu Wang, Yang Luo, Pin Zhang, Qing Li, Binghe Xu^*^

Correspondence to: xubinghe@medmail.com.cn

**This PDF file includes:**

Materials and Methods.

Supplementary Table 1. The demographic and clinical characteristics of enrolled patients at baseline.

Supplementary Fig 1. Study flowchart.

Supplementary Fig 2. Kaplan-Meier estimates of progression-free survival and overall survival for patients receiving pyrotinib plus capecitabine and lapatinib plus capecitabine according to prior trastuzumab treatment.

List of abbreviations.

**Materials and methods**

**Study design and population**

Individual patient data was collected and summarized from the phase I trial for pyrotinib plus capecitabine (NCT02361112), the pivotal phase II trial (NCT02422199) and the PHOEBE phase III trial (NCT03080805) that enrolled patients in the National Cancer Center of China. In the above three trials, female patients of HER2-positive metastatic breast cancer were assigned to receive pyrotinib 400 mg or lapatinib 1250 mg orally once per day for 21-day cycles, both combined with capecitabine (1000 mg/m^2^ orally twice per day on days 1 to 14).

Each of the patients enrolled in these studies were aged between 18 and 70 years, had a pathologically confirmed diagnosis of HER2-positive (defined as either an immunohistochemistry score of 3+ or 2+ together with HER2 gene amplification by fluorescence in situ hybridization) relapsed or metastatic breast cancer, had been previously treated with taxanes, had at least one measurable lesion defined by revised Response Evaluation Criteria in Solid Tumors guidelines version 1.1 (RECIST 1.1), had a Eastern Cooperative Oncology Group (ECOG) performance status of 0 or 1, and had adequate bone marrow and organ function. Major exclusion criteria were previous treatment with anti-HER2 tyrosine kinase inhibitors (TKIs), previous ineffective standard capecitabine treatment (ie, disease progression during capecitabine treatment or a response lasting less than 3 months after capecitabine treatment discontinuation), previous effective standard capecitabine treatment less than 6 months before enrollment, any anticancer treatment given within 4 weeks before enrollment or history of brain metastasis.

The protocol was approved by the Institutional Review Board of National Cancer Center / Cancer Hospital Chinese Academy of Medical Sciences and Peking Union Medical College. This study was conducted in accordance with the International Conference on Harmonization Guideline for Good Clinical Practice and the ethical principles in the Declaration of Helsinki. Each of the patients provided written informed consent before participation. The study protocol was approved by all participating investigators.

**Procedures and outcomes**

Original individual patient-level data was collected and analyzed regarding clinicopathological characteristics, treatment, clinical outcomes and updated survival in this analysis. To reduce potential bias, the information was verified for all the enrolled patients including those who had been excluded from the investigators in original analyses.

Outcome measures in this analysis were progression-free survival and overall survival. Progression-free survival was defined as the time from the date of enrollment to the date of first documentation of disease progression confirmed by blinded independent central review or date of death due to any cause, whichever occurred first. Overall survival was defined as the time from the date of enrollment to the date of death due to any cause.

**Statistical analysis**

All the patients recruited in these trials from the National Cancer Center of China were considered assessable and were included in this pooled dataset. The clinicopathological characteristics of the recruited patients were described in percentages of categorical variables. χ2 test or Fisher’s test were used to compare the distribution of clinicopathological characteristics between different groups. Kaplan-Meier estimations were utilized to report outcomes of PFS and OS, and a stratified Cox proportional hazards model was used to estimate hazard ratios (HR) and 95% confidence intervals (CI). All statistical tests were two-sided, and P values below 0.05 were considered statistically significant. All analyses were performed with SPSS software (version 23.0, SPSS Inc., Chicago, IL, USA). Kaplan-Meier survival plots were performed, and the number at risk was determined using MedCalc (version 20.022, MedCalc Software Bvba, Ostend, Flanders).

Supplementary Table 1. The demographic and clinical characteristics of enrolled patients at baseline

|  | Pyrotinib plus capecitabine group (n=36) | Lapatinib plus capecitabine group (n=29) | *χ^2^* | *P* value |
| --- | --- | --- | --- | --- |
| Age |  |  | / | 1.000 |
| <60 | 31(86.1) | 25(86.2) |  |  |
| ≥60 | 5(13.9) | 4(13.8) |  |  |
|  |  |  |  |  |
| Menstrual status |  |  | 0.979 | 0.323 |
| Premenopausal | 23(63.9) | 15(51.7) |  |  |
| Postmenopausal | 13(36.1) | 14(48.3) |  |  |
|  |  |  |  |  |
| ER status |  |  | 0.979 | 0.323 |
| Positive | 13(36.1) | 14(48.3) |  |  |
| Negative | 23(63.9) | 15(51.7) |  |  |
|  |  |  |  |  |
| PR status |  |  | 0.042 | 0.839 |
| Positive | 14(38.9) | 12(41.4) |  |  |
| Negative | 22(61.1) | 17(58.6) |  |  |
|  |  |  |  |  |
| Tumor site |  |  | / | 0.742 |
| Visceral | 29(80.6) | 25(86.2) |  |  |
| Non-visceral | 7(19.4) | 4(13.8) |  |  |
|  |  |  |  |  |
| Prior taxane treatment, n (%) | 36(100.0) | 29(100.0) | / | / |
|  |  |  |  |  |
| Prior anthracycline treatment, n (%) | 31(86.1) | 26(89.7) | / | 0.723 |
|  |  |  |  |  |
| Prior trastuzumab treatment, n (%) |  |  | 1.293 | 0.731 |
| Trastuzumab-pretreated for metastatic disease only | 13(36.1) | 7(24.1) |  |  |
| Trastuzumab-pretreated in the adjuvant/neoadjuvant setting only | 12(33.3) | 11(37.9) |  |  |
| Trastuzumab-pretreated in both adjuvant/neoadjuvant setting and metastatic setting | 5(13.9) | 4(13.8) |  |  |
| Non | 6(16.7) | 7(24.1) |  |  |


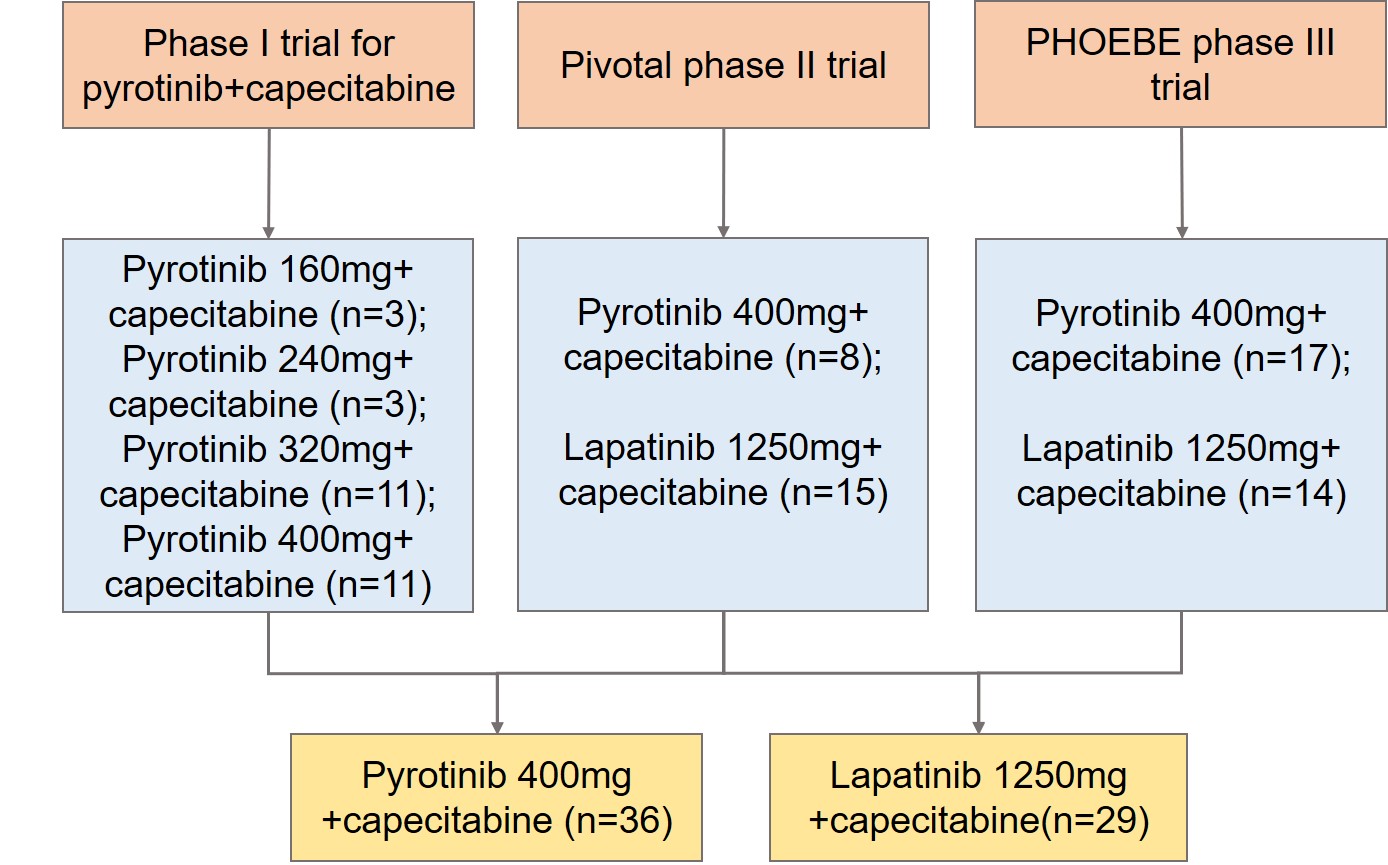


Supplementary Fig 1. Study flowchart.


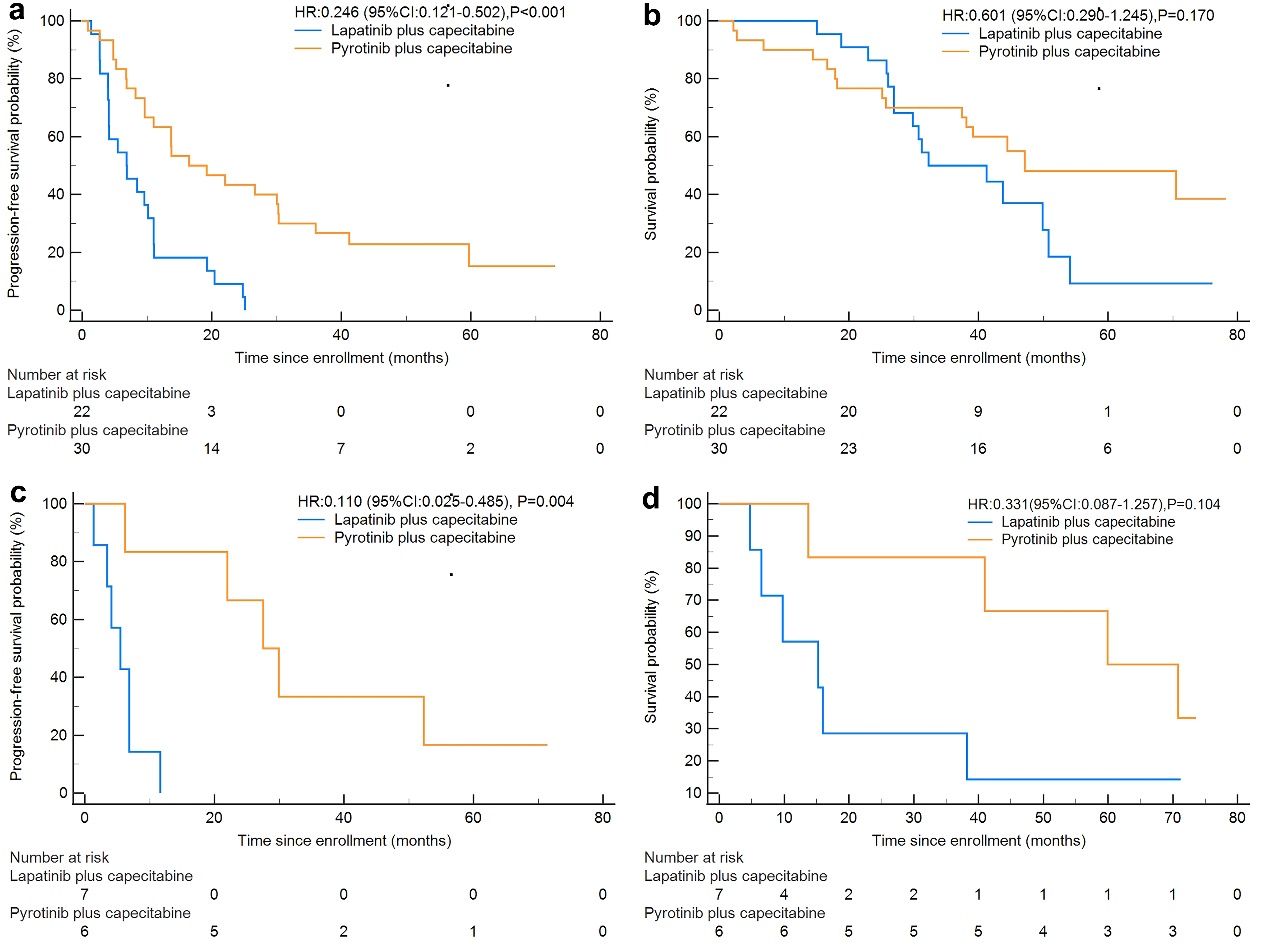


Supplementary Fig 2. Kaplan-Meier estimates of progression-free survival and overall survival for patients receiving pyrotinib plus capecitabine and lapatinib plus capecitabine according to prior trastuzumab treatment.

Kaplan-Meier estimates of (a) progression-free survival and (b) overall survival for patients receiving pyrotinib plus capecitabine and lapatinib plus capecitabine in those who were previously treated with trastuzumab.

Kaplan-Meier estimates of (c) progression-free survival and (d) overall survival for patients receiving pyrotinib plus capecitabine and lapatinib plus capecitabine in those trastuzumab-naive patients.

**List of abbreviations**

HER2, human epidermal growth factor receptor 2;

EGFR, epidermal growth factor receptor;

TKI, tyrosine kinase inhibitor;

PFS, progression-free survival;

MBC, metastatic breast cancer;

L+C, lapatinib plus capecitabine;

P+C, pyrotinib combined with capecitabine, pyrotinib plus capecitabine

OS, overall survival;

HR, hormone receptor;

EGFR, epidermal growth factor receptor;

ADCs, Antibody-Drug Conjugates.
