# Supplementary figures and images for: Pyrotinib plus capecitabine could significantly improve overall survival in HER2-positive metastatic breast cancer
Source: Signal Transduct Target Ther. 2023 Mar 19;8:118. doi: 10.1038/s41392-023-01322-w (PMC10025258; doi:10.1038/s41392-023-01322-w)

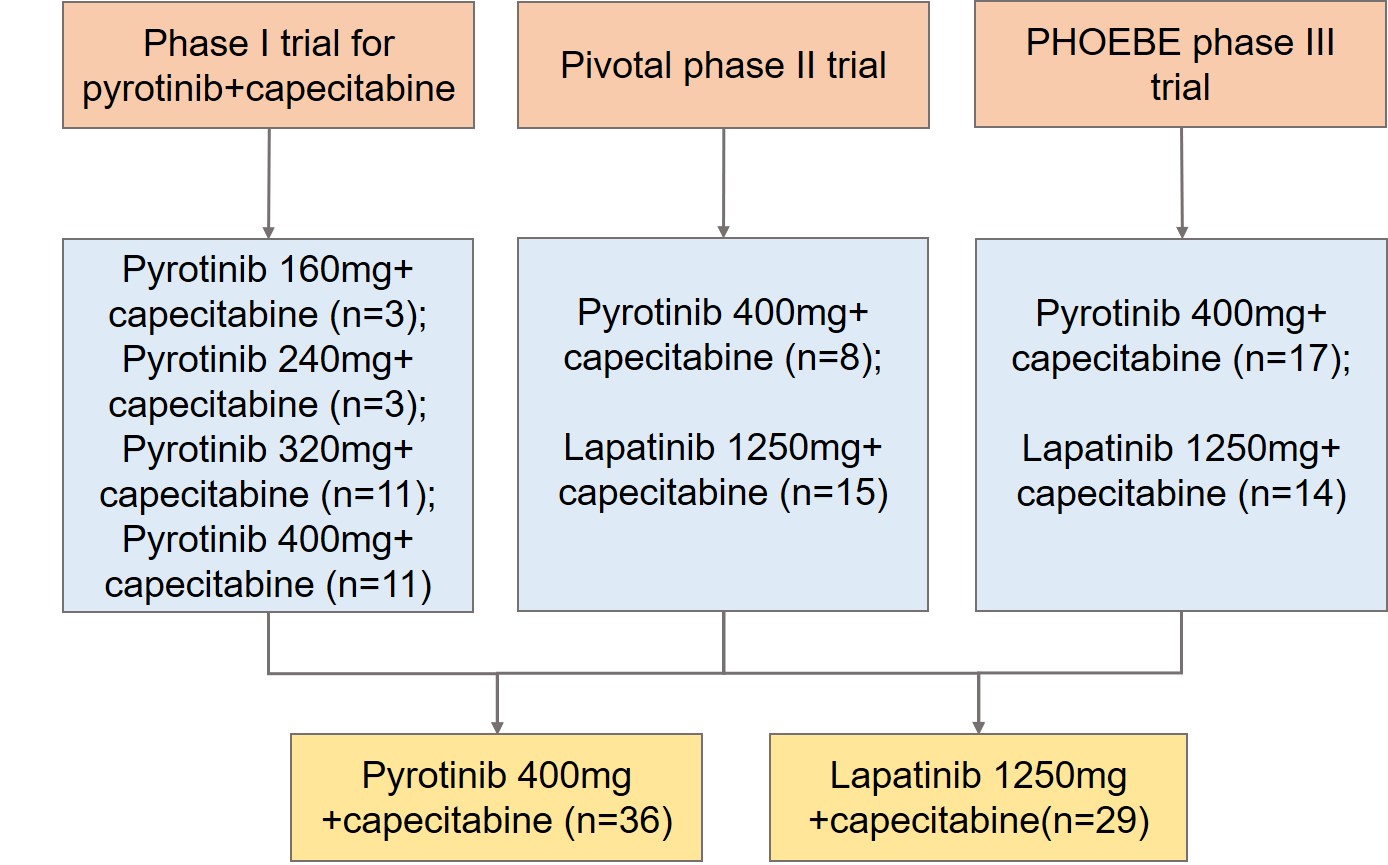

Supplement: Supplementary file 2 — Supplementary Figure 1 [file 41392_2023_1322_MOESM2_ESM.jpg]

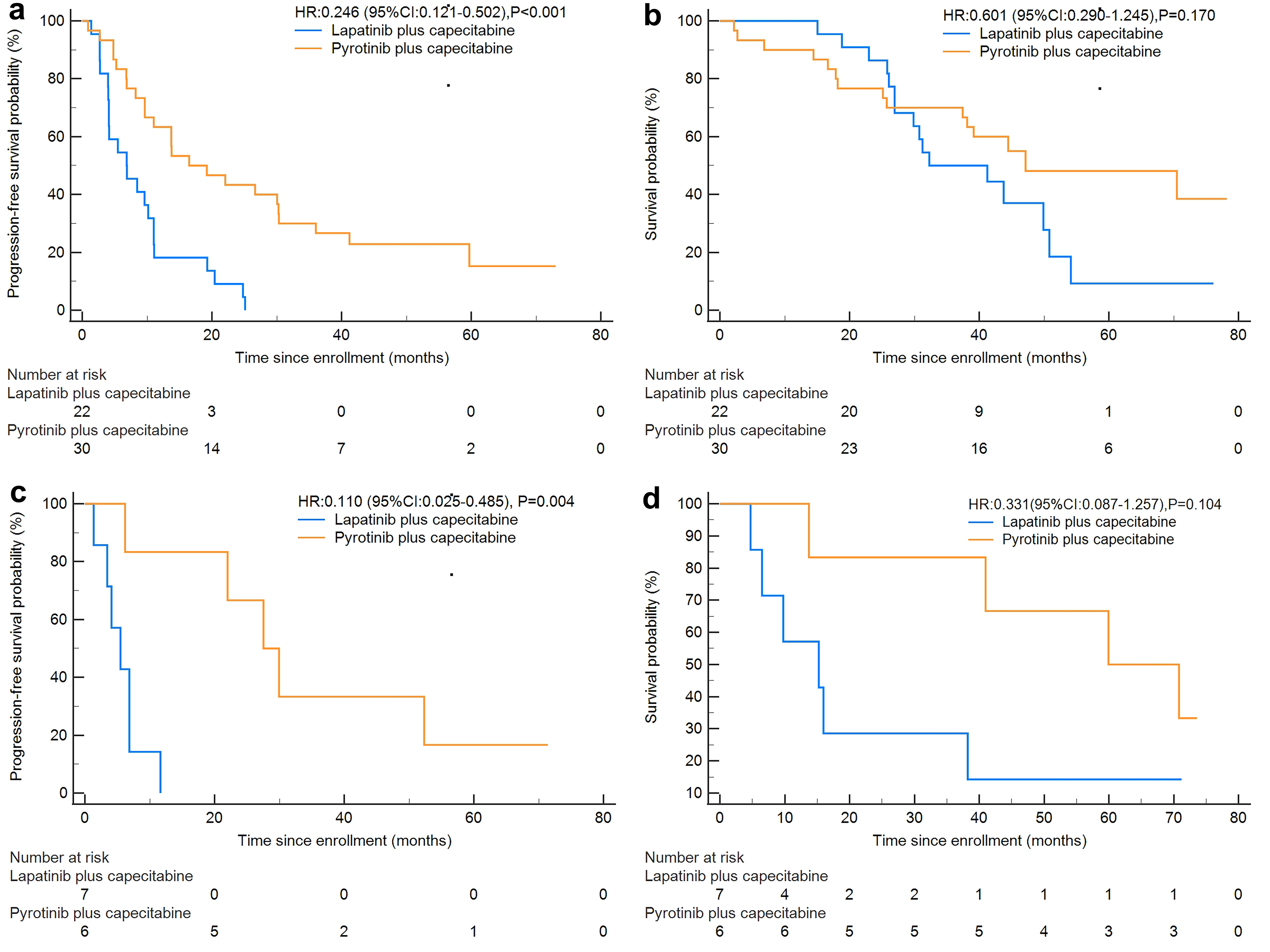

Supplement: Supplementary file 3 — Supplementary Figure 2 [file 41392_2023_1322_MOESM3_ESM.jpg]
